# Functional Similarities between Pigeon ‘Milk’ and Mammalian Milk: Induction of Immune Gene Expression and Modification of the Microbiota

**DOI:** 10.1371/journal.pone.0048363

**Published:** 2012-10-26

**Authors:** Meagan J. Gillespie, Dragana Stanley, Honglei Chen, John A. Donald, Kevin R. Nicholas, Robert J. Moore, Tamsyn M. Crowley

**Affiliations:** 1 Australian Animal Health Laboratory, CSIRO Animal, Food and Health Sciences, Geelong, Victoria, Australia; 2 School of Life and Environmental Sciences, Deakin University, Geelong, Victoria, Australia; 3 Centre for Chemistry and Biotechnology, Deakin University, Geelong, Victoria, Australia; INRA, UR1282, France

## Abstract

Pigeon ‘milk’ and mammalian milk have functional similarities in terms of nutritional benefit and delivery of immunoglobulins to the young. Mammalian milk has been clearly shown to aid in the development of the immune system and microbiota of the young, but similar effects have not yet been attributed to pigeon ‘milk’. Therefore, using a chicken model, we investigated the effect of pigeon ‘milk’ on immune gene expression in the Gut Associated Lymphoid Tissue (GALT) and on the composition of the caecal microbiota. Chickens fed pigeon ‘milk’ had a faster rate of growth and a better feed conversion ratio than control chickens. There was significantly enhanced expression of immune-related gene pathways and interferon-stimulated genes in the GALT of pigeon ‘milk’-fed chickens. These pathways include the innate immune response, regulation of cytokine production and regulation of B cell activation and proliferation. The caecal microbiota of pigeon ‘milk’-fed chickens was significantly more diverse than control chickens, and appears to be affected by prebiotics in pigeon ‘milk’, as well as being directly seeded by bacteria present in pigeon ‘milk’. Our results demonstrate that pigeon ‘milk’ has further modes of action which make it functionally similar to mammalian milk. We hypothesise that pigeon ‘lactation’ and mammalian lactation evolved independently but resulted in similarly functional products.

## Introduction

Pigeon ‘milk’ is a substance produced in the crop of both male and female pigeons for the nourishment of their young. Similarly, male and female flamingos [1] and male emperor penguins [2] can produce crop ‘milk’, but there is a paucity of information available about these processes. Like mammalian lactation, pigeon ‘milk’ production is regulated by the lactogenic hormone prolactin [3]. The resulting pigeon crop ‘milk’ consists of lipid-filled, protein rich keratinocytes that have proliferated and separated from the germinal epithelium of the crop sac to form a curd-like substance that is regurgitated to the squab [4]. This cheesy substance also contains bacteria [5]. Like mammalian milk, pigeon ‘milk’ is highly nutritious, consisting of protein (60%), fat (32–36%), carbohydrate (1–3%) and minerals (calcium, potassium, sodium and phosphorus) [6]; it also contains IgA antibodies [7]. Interestingly, if squabs are fed a nutritional replacement of pigeon ‘milk’ they die or fail to thrive [8], which suggests that there are factors aside from nutrition in pigeon ‘milk’ that influence development of the young. Like mammalian milk components, these factors in pigeon ‘milk’ may play a role in immune development. Mammalian milk can modulate the development of the immune system directly, by delivering immune molecules such as immunoglobulins and cytokines [9], [10], and indirectly by influencing the microbiota through prebiotics [11].

The bacterial composition of the gut of breast fed infants is very different to formula fed infants, as it is influenced by prebiotics in the breast milk [12]. Similarly, the gut microbial composition of mother-fed piglets differs to formula-fed piglets [13]. These differences in microbiota are significant as it has been shown that the gut microflora of the developing infant can play a role in the developing immune system [14] and in energy and nutrient capture [15]. The first contact between the immune system and the gut microflora is by the Gut Associated Lymphoid Tissue (GALT), which comprises the largest lymphoid tissue mass in the human body [16]. The GALT is also the largest site of IgA production in the body, synthesising over 60% of all IgA produced [16]. Development of IgA B cells is dependent on microbial colonisation [17], and consequently, colostrum contains high levels of IgA [9], as the infant has not yet established a microbiome to facilitate production of IgA.

Not only does mammalian milk modulate the microbiota of the developing infant and provide copious amounts of IgA, it also contains a gamut of other immune modulators that contribute to the immune protection of the immunologically naive infant by either modulating development of the immune system or providing passive immunity [18]. At birth, the human infant is deficient in certain cytokines and cells of the myeloid lineage, and others have impaired function [19], which renders the infant reliant on maternal passive immunity and on milk components that aid in the development of the immune system. These components include cytokines, chemokines and colony stimulating factors [20], as well as maternally-derived immune cells [21], [22]. A breast fed human infant consumes an estimated 10^8^ immune cells per day, which consist of 55–60% macrophages, 30–40% neutrophils and 5–10% lymphocytes [21], [22]. Other beneficial substances found in milk include hormones such as epidermal growth factor [23], [24], enzymes such as lysozyme (which also has antimicrobial activity) [25], and other antimicrobial proteins such as lactoferrin [26], [27].

Pigeon ‘milk’ has been shown to contain a number of bioactive proteins including IgA [7], a pigeon ‘milk’ growth factor with biological activity similar to epidermal growth factor [28], [29], and transferrin [30], a glycoprotein with a similar sequence and structure to lactoferrin [31]. In addition, it has been shown that chickens fed pigeon ‘milk’ had a higher rate of growth than chickens not receiving pigeon ‘milk’ [32], [33], which could be attributed to the increased caloric intake and/or the beneficial effect of bacteria and bioactive molecules in pigeon ‘milk’. However, there have been no studies explicitly examining whether pigeon ‘milk’ can modulate immune tissues. Previous studies in chickens have shown that bacteria is important for the development of the GALT [34]. Here we test the hypothesis that pigeon ‘milk’ will alter the intestinal microbiota and effect expression of genes in the GALT. We show that pigeon ‘milk’-fed chickens had a different microbial composition in their caeca to control chickens, and they also showed significant enrichment of immune-related genes among genes differentially expressed in GALT tissues.

## Results

### Chickens fed pigeon ‘milk’ had increased body mass

At the start of the experiment (day 0) and at day 4, there was no significant difference between the body mass of pigeon ‘milk’ (PM)-fed chickens and control chickens (Table 1). After 7 days, PM-fed chickens had grown on average 12.5% heavier than control chickens. A nutritional replacement of pigeon ‘milk’ had no effect on the growth of chickens compared to the control group (Figure S1). Interestingly, the breast muscle made up a significantly (*p*<0.05) higher proportion of total body mass in the PM-fed chickens and the wing span of PM-fed chickens was wider compared to normally fed chickens (Table 1). The leg span of PM-fed chickens tended to be wider (p = 0.0558; Table 1) as did the height (p = 0.0820; Table 1). This increase in size was also accompanied by a decrease in feed conversion ratio (FCR); PM-fed chickens had an average FCR of 1.34 compared to 1.47 for control chickens (Table 1).

**Table 1:** Comparison of chicken body measurements by group.

Measurement	Control (n = 8)	PM-fed (n = 8)	*p* value
Day 0 body mass	43.14 g±1.024 g	41.90 g±1.647 g	0.2672
Day 4 body mass	67.63 g±2.337 g	72.25 g±3.807 g	0.1590
Day 7 body mass	137.0 g±7.530 g	154.2 g±5.467 g	0.0426*
Breast muscle mass	6.793 g±0.6869 g	9.289 g±0.7624 g	0.0145*
Proportion of breast muscle to body mass	4.868±0.3180	5.973±0.3780	0.0210*
Height	14.75 cm±0.2113 cm	15.19 cm±0.2100 cm	0.0820
Wing span	7.563 cm±0.1752 cm	8.000 cm±0.1336 cm	0.0335*
Leg span	8.850 cm±0.1615 cm	9.219 cm±0.1451 cm	0.0558

Body measurements of control and PM-fed chickens were analysed statistically using an unpaired t-test and the results are presented as the mean ± standard deviation.

* significantly different (*p*<0.05)

### Pigeon ‘milk’ affected gene expression in the GALT

Differential gene expression in the GALT was analysed using tissue from ileum and caecal tonsil because they contain a high proportion of GALT. A comparison of gene expression in the ileum of PM-fed chickens to control chickens revealed 2202 differentially expressed genes (*p*<0.05); 1586 of these genes were up-regulated and 616 were down-regulated. In addition, a comparison of gene expression in the caecal tonsil of PM-fed chickens to control chickens revealed 1131 differentially expressed genes (*p*<0.05); 522 of these genes were up-regulated and 609 were down-regulated.

**Figure 1:**
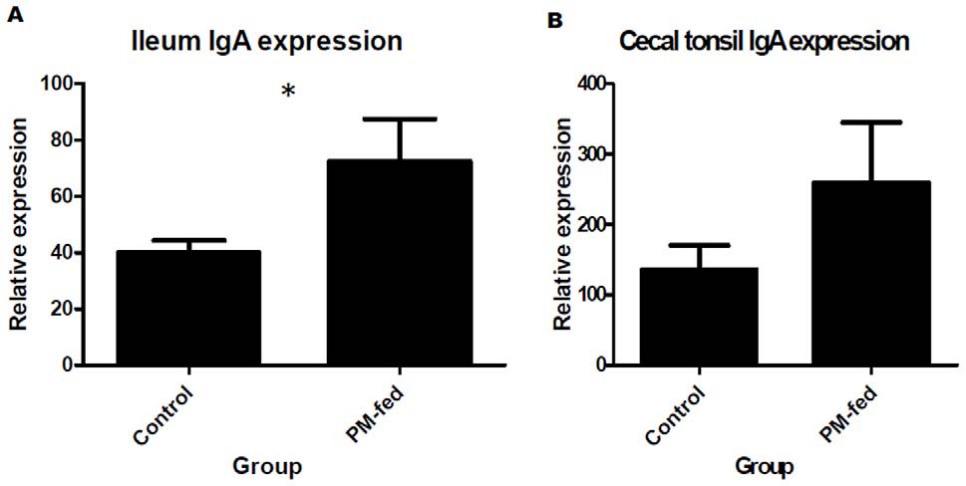
IgA mRNA expression in the GALT. Expression of IgA heavy chain mRNA was significantly higher in PM-fed chickens in the ileum (p = 0.033) and also tended to be higher in the caecal tonsil (p = 0.11), as compared to control chickens.

Functional analysis of the up-regulated genes by gene ontology in PM-fed chickens identified four immune-specific gene ontology biological processes in the ileum and 23 in the caecal tonsil (Table S1). Regulation of B cell activation was enriched in both ileum and caecal tonsil (Table S1) and analysis of the transcription of IgA heavy chain (transcribed in B cells) revealed that PM-fed chickens had a significantly higher level of IgA expression than control chickens in the ileum (*p*<0.05), and a trend toward higher expression in the caecal tonsil (*p* = 0.1265) (Figure 1). There were no immune-specific gene ontology biological processes down-regulated in either the ileum or caecal tonsil (Table S2). Down-regulated GO biological processes in the ileum related to cell cycle control and apoptosis, and lipid synthesis and metabolism in the caecal tonsil (Table S2). Three up-regulated immune-specific KEGG pathways were identified in the ileum and only one in the caecal tonsil. There were no down-regulated KEGG pathways in the ileum. In the caecal tonsil, there were two down-regulated KEGG pathways related to the splicesome and the actin cytoskeleton (Table S3).

Six interferon-stimulated genes (ISGs) were up-regulated in the ileum and ten in the caecal tonsil (Table 2). The majority of these ISGs relate to host defence (five), antiviral (five), transcription factor or activator (four) or immune modulation (three) (Table 2).

**Table 2:** Interferon stimulated genes up-regulated in the gut of PM-fed chickens.

Gene	Functional classification	Probe name	*p* value	Fold change
**Ileum**				
similar to complement component C2	ComplementImmune modulation	RIGG20413	0.009	1.25
Fibroblast growth factor 2 (basic)	AngiogenesisDevelopmentGrowth factor	RIGG16507	0.015	1.21
		CLIGg_41549	0.049	1.17
Macrophage stimulating 1 (hepatocyte growth factor-like)	Growth factorSignaling	CLIGg_00552	0.009	2.15
		RIGG07902	0.003	2.14
Interferon regulatory factor 7	Host defenseTranscription factorTranscriptional activator	RIGG17886	0.019	1.53
		CLIGg_00887	0.023	1.38
Interferon regulatory factor 1	Host defenseImmune modulationSignalingTranscription factorTranscriptional activator	CLIGg_00658	0.035	1.34
Interferon regulatory factor 4	OncogeneTranscription factorTranscriptional activator	RIGG09155	0.032	1.24
**Caecal tonsil**				
Interferon-induced protein with tetratricopeptide repeats 5	Unknown	CLIGg_28648	0.006	2.76
		RIGG13336	0.010	2.52
		RIGG07326	0.006	2.47
Myxovirus (influenza virus) resistance 1, interferon-inducible protein p78 (mouse)	AntiviralGTP-bindingHost defense	RIGG18960	0.005	2.20
		Misc_00001	0.005	1.97
2′-5′-oligoadenylate synthetase-like	AntiviralHost defense	CLIGg_00435	0.019	2.17
		RIGG01751	0.045	1.94
Fibrinogen gamma chain	Blood clotting	RIGG14995	0.031	1.69
Beta-2-microglobulin precursor	Antigen presentationHost defense	RIGG10931	0.009	1.35
Interferon induced with helicase C domain 1 (MDA5)	Apoptosis [61]Antiviral [62], [63]	RIGG16089	0.033	1.30
		RIGG07546	0.029	1.24
		Misc_00005	0.042	1.23
Zinc finger CCCH-type, antiviral 1 (ZAP)	Antiviral [64]	RIGG19894	0.010	1.22
Similar to interferon-induced membrane protein 1 (IFITM1)	Antiviral [65], [66]	CLIGg_06123	0.003	1.21
		RIGG12134	0.005	1.19
Complement component 1, q subcomponent, C chain	ComplementImmune modulation	CLIGg_08804	0.047	1.20
V-ets erythroblastosis virus E26 oncogene homolog 2 (avian)	DevelopmentTranscription factorTranscriptional activator	CLIGg_04698	0.014	1.10

Genes up-regulated in PM-fed chicken (n = 6) gut which are known interferon-stimulated genes. No known interferon-stimulated genes were down-regulated.

### Pigeon ‘milk’ influenced bacterial diversity and abundance

Statistical analysis of comparative abundance of bacteria between control and PM-fed chickens revealed that the PM had caused very significant changes in the population structure of the caecal microflora of PM-fed chickens. Many groups of bacteria were differentially abundant between control and PM-fed chickens at the levels of phylum, class, order, family and genus. Comparative analysis of bacterial abundance at a phylum level (Table 3) showed that bacterial 16S sequences were assigned to three bacterial phyla, and bacterial abundance was statistically different between control and PM-fed chickens in one of these phyla (Proteobacteria). The most abundant phyla in both groups was Firmicutes, constituting 99.622% of all control chicken bacteria and 96.630% of PM-fed bacteria, which had a more diverse range of bacteria (Table 3). The remainder of PM-fed bacteria belonged to Proteobacteria (0.318%) or were unassigned (3.052%). PM-fed chickens had no detected Bacteroidetes, whereas control chickens had 0.003% Bacteroidetes, and the remaining were Proteobacteria (0.021%) and unassigned (0.354%).

**Table 3:** Proportions of bacterial phyla present in control and PM-fed chickens.

Classification	Control (%) (n = 8)	PM-fed (%) (n = 8)	*p* value
**Phylum**			
Bacteroidetes	0.003±0.003	0.000±0.000	1
Firmicutes	99.622±0.234	96.630±1.705	0.082
Proteobacteria	0.021±0.009	0.318±0.126	0.004*
Unassigned	0.354±0.238	3.052±1.634	0.120
**Class**			
Bacilli	77.117±5.666	57.917±6.345	0.022*
Bacteroidia	0.003±0.003	0.000±0.000	1
Betaproteobacteria	0.000±0.000	0.315±0.127	0.013*
Clostridia	22.026±5.323	37.378±5.452	0.045*
Erysipelotrichi	0.059±0.031	0.088±0.051	0.551
Gammaproteobacteria	0.021±0.009	0.003±0.003	0.068
Unclassified	0.775±0.483	4.298±1.564	0.030*
**Order**			
Bacillales	0.269±0.216	0.096±0.055	0.441
Bacteroidales	0.003±0.003	0.000±0.000	1
Burkholderiales	0.000±0.000	0.315±0.127	0.013*
Clostridiales	22.026±5.323	37.312±5.426	0.040*
Enterobacteriales	0.021±0.0090	0.003±0.003	0.059
Erysipelotrichales	0.059±0.031	0.088±0.051	0.596
Lactobacillales	76.848±5.791	57.821±6.373	0.023*
Unclassified	0.775±0.483	4.364±1.556	0.024*
**Family**			
Alcaligenaceae	0.000±0.000	0.315±0.127	0.013*
Bacillaceae	0.269±0.216	0.096±0.055	0.472
Bacteroidaceae	0.003±0.003	0.000±0.000	1
Enterobacteriaceae	0.021±0.009	0.003±0.003	0.068
Enterococcaceae	0.464±0.32	1.802±0.381	0.007*
Erysipelotrichaceae	0.059±0.031	0.088±0.051	0.644
Eubacteriaceae	0.065±0.034	0.043±0.026	0.625
Incertae Sedis XIII	0.012±0.009	0.008±0.006	0.710
Incertae Sedis XIV	0.017±0.007	0.007±0.007	0.329
Lachnospiraceae	7.643±2.45	9.208±1.704	0.622
Lactobacillaceae	76.179±5.923	55.262±6.423	0.017*
Peptostreptococcaceae	5.21±1.894	1.14±0.273	0.039*
Ruminococcaceae	7.367±1.2	9.3±1.656	0.370
Streptococcaceae	0.144±0.069	0.527±0.489	0.471
Unclassified	2.546±0.733	20.59±4.746	0.001*
Veillonellaceae	0.000±0.000	1.611±0.999	0.111
**Genus**			
*Anaerotruncus*	1.725±0.583	1.555±0.251	0.781
*Bacteroides*	0.003±0.003	0.000±0.000	1
*Blautia*	0.017±0.007	0.007±0.007	0.344
*Butyricicoccus*	0.323±0.162	0.419±0.138	0.660
*Enterococcus*	0.464±0.32	1.802±0.381	0.008*
*Escherichia/Shigella*	0.021±0.009	0.003±0.003	0.058
*Eubacterium*	0.065±0.034	0.043±0.026	0.613
*Faecalibacterium*	0.113±0.103	0.657±0.439	0.264
*Lactobacillus*	70.772±6.107	52.351±6.442	0.037*
*Oscillibacter*	0.194±0.107	0.371±0.083	0.219
*Roseburia*	0.119±0.048	0.23±0.107	0.378
*Sporacetigenium*	5.194±1.897	1.14±0.273	0.034*
*Streptococcus*	0.144±0.069	0.527±0.489	0.470
*Subdoligranulum*	0.000±0.000	0.037±0.023	0.120
*Sutterella*	0.000±0.000	0.315±0.127	0.015*
*Unclassified*	20.846±4.02	38.93±6.31	0.017*
*Veillonella*	0.000±0.000	1.611±0.999	0.113

The proportion of bacteria present in each phylum, by chicken group. Proportional abundance of bacteria in each phylum was calculated using Metastats and the results are presented as the mean ± the standard error.

* *p*<0.05

At the class level bacteria from the two groups of chickens were classified into 6 classes (Table 3); three of which were significantly differentially abundant between PM-fed and control chickens (Bacilli, Betaproteobacteria, and Clostridia) (Table 3). Bacilli was the most abundant class of bacteria in both groups of chickens (77.117% in control chickens and 57.917% in PM-fed chickens) followed by Clostridia (22.026% in control chickens and 37.378% in PM-fed chickens) (Table 3). At the order level, there were three bacterial orders significantly differentially abundant between PM-fed and control chickens out of seven orders classified (Table 3). These were Burkholdierales (not present in control chickens and 0.315% in PM-fed), Clostridiales (22.026% in controls and 37.312% in PM-fed) and Lactobacillales (76.848% in controls and 57.821% in PM-fed) (Table 3).

16S sequences from both chicken groups were assigned to 15 families, four of which were significantly differentially abundant (Table 3). These were Alcaligenaceae (not present in control and 0.315% of PM-fed), Enterococcaceae (0.464% of control and 1.802% of PM-fed), Lactobacillaceae (76.179% of control and 55.262% PM-fed) and Peptostreptococcaceae (5.21% of control and 1.14% of PM-fed) (Table 3). In addition to Alcaligenaceae, control chickens had no Veillonellaceae (PM-fed 1.611%) (Table 3). Conversely, PM-fed chickens had no Bacteroidaceae (control 0.003%) (Table 3).

At the genus level, sequences were classified into 16 genera, four of which were significantly differentially abundant (Table 3). These were *Enterococcus* (control 0.464%, PM-fed 1.802%), *Lactobacillus* (control 70.772%, PM-fed 52.351%), *Sporacetigenium* (control 5.194%, PM-fed 1.14%), and *Sutterella* (control not present, PM-fed 0.315%) (Table 3). In addition to *Suterella*, control chickens had no *Veillonella* (PM-fed 1.611%) or *Subdoligranulum* (PM-fed 0.037%) (Table 3). PM-fed chickens had no *Bacteroides* (control 0.003%) (Table 3).

### PM-fed chickens shared a number of bacteria present in PM

**Figure 2:**
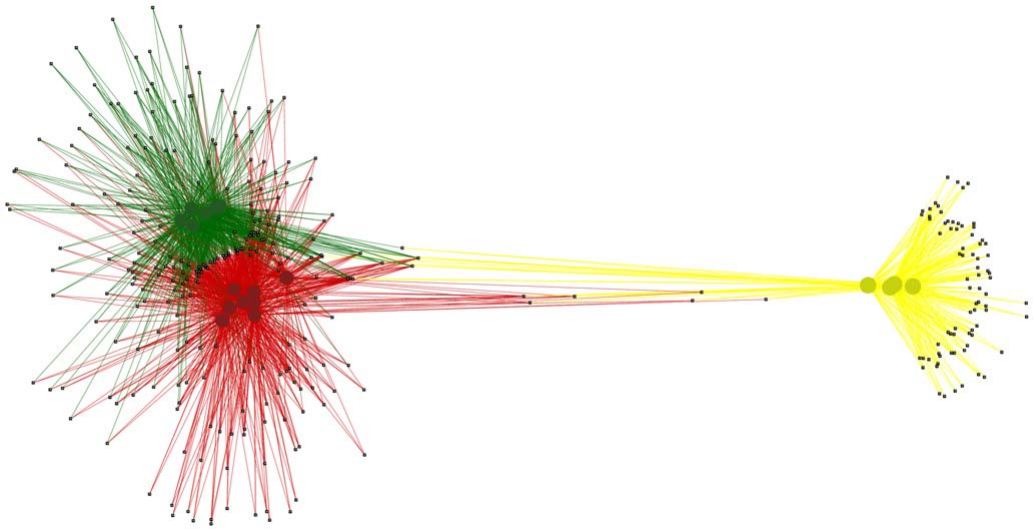
Network analysis of OTUs present in PM, PM-fed chickens and control chickens. PM-fed chickens (large red circles) and control chickens (large green circles) form distinct groups based on OTU (small black squares) abundance, although they still share many OTUs. PM (large yellow circles) was distinct from both groups of chickens. PM-fed chickens and PM shared six OTUs that were not present in control chickens. There were eight OTUs shared by all three groups. PM and control chickens shared only one OTU that was not present in PM-fed chickens.

Network analysis of Operational Taxonomic Units (OTUs) shared between groups (Figure 2) revealed that PM-fed chickens share several OTUs with PM that are not present in control chickens, and control chickens share only one OTU with PM that is not present in PM-fed chickens. Additionally, control chickens and PM-fed chickens share many OTUs that are not present in PM, but they cluster as distinct groups (Figure 2).

**Figure 3:**
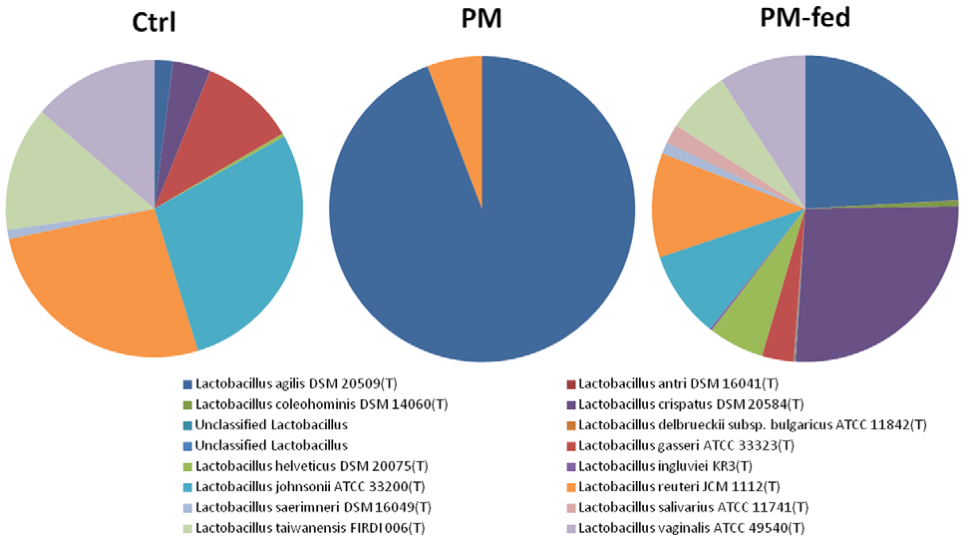
Proportion of *Lactobacillus* species present in PM, PM-fed chickens and control chickens. The genus *Lactobacillus* was represented by only 2 species of bacteria in PM, whereas control and PM-fed chickens had a greater number of species that constitute the total population of *Lactobacillus*. PM-fed chickens had a more diverse *Lactobacillus* population than control chickens (16 species and 12 species, respectively), and the species abundance as a proportion of the total *Lactobacillus* population was also very different between the two groups.

Analysis of the six OTUs present only in PM and PM-fed chickens revealed that four of the six OTUs are most closely related to *Veillonella* species (*V. criceti, V. caviae, V. magna and V. ratti*), one was identified as *Enterococcus columbae*, and one was most closely related to *Sutterella stercoricanis* (Table 4). The one OTU that was shared by PM and control chickens was most closely related to *Bacteroides paurosaccharolyticus*, and was present in very low abundance in control chickens (Table 4). The eight OTUs that are shared between all three groups were all identified as two *Lactobacillus* species; *L. reuteri* and *L. agilis* (Table 4). Analysis of the total Lactobacillus population in all groups (Figure 3) revealed that *L. agilis* and *L. reuteri* made up the entire *Lactobacillus* population of PM (94.19% and 5.82% respectively). *L. agilis* constituted 24.11% of PM-fed chicken *Lactobacillus*, whereas it constituted only 2.01% of control chicken *Lactobacillus* (Figure 3). *L. reuteri* constituted a higher percentage of control chicken *Lactobacillus* (26.47%) than PM-fed chicken *Lactobacillus* (11.01%), whereas PM-fed chicken total *Lactobacillus* had a higher proportion of *L. crispatus* and *L. helveticus* (26.23% and 5.89% respectively) than control chickens (4.10% and 0.38% respectively) (Figure 3). The PM-fed chicken total *Lactobacillus* population was more diverse than in control chickens, with 16 *Lactobacillus* species present compared to 12 in control chickens (Figure 3). The four species not present in control chickens make up a small percentage of the total PM-fed *Lactobacillus* population (*L. coleohominis* 0.62%, *L. delbruckii subsp. Bulgaricus* 0.09%, *L. ingluvei* 0.24% and *L. salivarius* 2.02%) (Figure 3).

**Table 4:** OTUs shared with PM.

OTU	Closest cultured isolate	Similarity(%)	Rarefied abundance		
			PM (n = 4)	PM-fed (n = 8)	Ctrl (n = 8)
**Present in PM and PM-fed chickens only**					
17	*Veillonella criceti ATCC 17747(T)*	94.41	33.53	35.95	0.00
86	*Sutterella stercoricanis CCUG 47620(T)*	95.42	2.27	7.64	0.00
88	*Veillonella caviae DSM 20738(T)*	94.82	18.44	0.91	0.00
183	*Enterococcus columbae LMG 11740(T)*	98.954	3.52	0.19	0.00
203	*Veillonella magna lac18(T)*	94.207	0.60	0.54	0.00
311	*Veillonella ratti DSM 20736(T)*	93.017	0.58	1.41	0.00
**Present in PM, PM-fed chickens and control chickens**					
3	*Lactobacillus reuteri JCM 1112(T)*	98.34	0.59	35.20	137.15
4	*Lactobacillus agilis DSM 20509(T)*	100	7.21	236.30	27.29
53	*Lactobacillus reuteri JCM 1112(T)*	99.349	0.10	51.00	241.19
97	*Lactobacillus agilis DSM 20509(T)*	99.554	0.51	11.06	1.14
107	*Lactobacillus agilis DSM 20509(T)*	98.718	0.11	6.58	0.25
217	*Lactobacillus agilis DSM 20509(T)*	99.111	0.10	4.27	0.26
334	*Lactobacillus agilis DSM 20509(T)*	98.95	1.02	39.41	2.31
393	*Lactobacillus agilis DSM 20509(T)*	97.976	0.25	17.46	6.60
**Present in PM and control chickens only**					
42	*Bacteroides paurosaccharolyticus WK042*	90.798	54.22	0.00	0.06

OTUs (bacterial identifiers) present in PM and another group were classified to their closest cultured isolate using EZTaxon. The rarefied abundance is mean number of times a bacteria was present in a random sampling of 1000.

## Discussion

This is the first study to investigate the effects of pigeon ‘milk’ on intestinal microbiota and gut gene expression. Our results demonstrate that, like mammalian milk, PM modulates the development of both the gut immune system and the gut microbiota. Pigeon ‘lactation’ and mammalian lactation, although produced by very different biological processes (one being a secretive process and the other a cellular exudate), have resulted in similarly functional products. Mammalian milk fulfils the needs of the developing young both nutritionally and immunologically. Here, we have shown that PM also appears to fulfil both these roles, as immune-related genes are significantly enriched in the gut of PM-fed chickens and there are significant differences between the microbiota of PM-fed chickens and control chickens.

A previous study found that pigeons fed a nutritional replacement of PM died or failed to thrive [8], so in order to make a comparison between newly hatched young that were fed PM and those that received a control diet, we used chickens, which are precocial and do not require any parental care. Previous studies have investigated the rate of growth of PM-fed chickens, reporting large increases in growth without any ill effects [32], [33]. Despite the great advances of the past decades in chicken breeding, which have provided massive gains in growth performance, the modern broiler chickens in our study still showed a significant improvement in growth when fed PM. A nutritional replacement of PM had no significant affect on chicken growth (Figure S1). PM-fed chickens had a 12.5% higher body mass than control chickens, but they were not significantly taller or with longer leg span (Table 1). Interestingly, there was an altered body composition, with the proportion of breast muscle to body mass significantly greater (23%) in PM-fed chickens (Table 1) which could suggest that the increased rate of growth is not only attributable to the slightly higher caloric intake of the PM-fed chickens. It could also be influenced by growth hormones such as Pigeon Milk Growth Factor (PMGF) [28] and/or bioactive molecules and bacteria in the PM.

This study has shown that, like mammalian milk, PM clearly influences the composition of the caecal microbiota. PM-fed chickens had a more diverse microbiota than control chickens at the level of phylum, class, order, family and genus (Table 3). Pigeon ‘milk’ could be a source of both probiotics and prebiotics. Three genera of bacteria were present in PM-fed chickens but not controls; *Subdoligranulum*, *Sutterella* and *Veillonella* (Table 3). Of these three genera, *Veillonella* and *Sutterella* were also present in PM but not control chickens (Table 4). Only one OTU, closest to the culturable isolate *Bacteroides paurosaccharolyticus*, was shared between control chickens and PM, but it was present in very low abundance in control chickens (0.06 as compared to 54.22 in PM)(Table 4), suggesting that the apparent absence in PM-fed chickens could simply be a depth of sampling issue.

Species of *Veillonella*, one of the two genera shared by PM and PM-fed chickens, has been characterised as having inhibitory activity against the enteropathogenic bacterial species *Listeria monocytogenes* [35], *Salmonella* Typhimurium [36], and *Salmonella* Enteritidis [37]. It is to that end that *Veillonella* is included in a probiotic product designed for poultry [38], which suggests that *Veillonella* species could be important probiotics in pigeon ‘milk’. All four of the *Veillonella* species shared by PM and PM-fed chickens have a 16S rRNA sequence divergence of more than 3% from the closest cultured isolate (Table 4), which suggests that the *Veillonella* species present in PM and PM-fed chickens could be novel species [39]. In addition, the *Sutterella* species shared by PM and PM-fed chickens is more than 3% divergent from the closest culturable isolate (Table 4), so it is also likely to be a novel species.

The variation in microbiota between PM-fed and control chickens and the relatively modest overlap in shared species between the PM and PM-fed chickens indicates that the PM is likely to be exerting its influence more by prebiotic effects rather than by the direct seeding of new microbiota. The presence of oligosaccharides in pigeon ‘milk’ [40] is indicative of one class of potential prebiotic. Composition of *Lactobacillus* populations varied greatly between groups, with PM-fed chickens having a more diverse *Lactobacillus* population than control chickens (Figure 3). This could be due to putative PM prebiotics, as there are many species of *Lactobacillus* that are amenable to the addition of prebiotics [41], [42]. In addition, there were more bacteria that were unclassified at the phylum level in PM-fed chickens (3.052%) than control chickens (0.354%) that could be potentially novel bacteria, some of which could be important in the functional modulation of the gut by PM.

Changes in gut microbiota can modulate the immune capabilities of the GALT, particularly by modulating IgA B cell development [17]. Consequently, the up-regulation of IgA heavy chain mRNA in the GALT of PM-fed chickens (Figure 1) and the up-regulation of various other genes implicated in immune processes (Table S1, Table S3) suggests that there could be modulation of the PM-fed chicken GALT by the microbiota. Gene ontology processes that were significantly enriched in GALT tissues of PM-fed chickens included the innate immune response, regulation of cytokine production and regulation of B cell activation and proliferation (Table S1), which are all suggestive of an immune effect of PM. Aside from the effect of microbiota, this could also be due to the effects of other as yet unidentified PM components such as cytokines and other bioactive peptides. In a study where chickens were given different bacterial inocula from chicken caeca, there was no up-regulation of any immune pathways or groups in the chicken GALT [43], which, aside from the differences in PM bacteria and chicken caecal bacteria, could suggest that PM modulates GALT development with immunomodulatory components that are in addition to the microbiota. Six ISGs are up-regulated in the ileum of PM-fed chickens, and ten in the caecal tonsil (Table 2). Four of these ISGs are also differentially expressed in breast-fed versus formula-fed infants [44]. In the chicken, these ISGs could have multiple interferon inducers from PM, including hormones. Two of the ISGs up-regulated in PM-fed chickens have been identified as targets of prolactin (interferon regulatory factor 1)[45] and the prolactin receptor (2′-5′-oligoadenylate synthetase)[46] which could suggest that, like mammalian milk [47], [48], PM production is not only induced by prolactin, but prolactin could be delivered to the young through the milk. Interestingly, four of the ISGs up-regulated in the caecal tonsil have antiviral activity (Table 2), which indicates PM may confer antiviral activity, which is again, functionally similar to mammalian milk [49], [50]. It is possible that the up-regulation of some of these immune genes is a response by the chicken to foreign antigens in the PM. However, the increase in body mass and bacterial diversity indicates PM is having a more beneficial effect on the chicken.

PM and mammalian milk both have nutritional and immune modulatory components, and the ability to modulate the microbiota of the gut. This is fascinating from an evolutionary point of view when one considers that mammals and birds evolved these processes independently. To this end, it would be interesting to investigate other bird species that have altricial young, as it may reveal additional ‘lactating’ bird species that were previously thought to be regurgitating seeds or insects to their young. This would allow comparative studies that could elucidate the evolutionary pressures that resulted in birds producing crop ‘milk’. Additionally, this would make for an interesting comparison with the evolutionary history of mammalian lactation.

## Conclusions

This study is the first to investigate the effects of pigeon ‘milk’ on the GALT and gut microbiota. Gene expression in the GALT of PM-fed chickens was significantly enriched with immune-related pathways, in particular ISGs, other components of the innate immune response, regulation of cytokine production and regulation of B cell activation and proliferation. The microbiota of PM-fed chickens was significantly more diverse than control chickens, and appears to be effected by prebiotics in pigeon ‘milk’, as well as being directly seeded by bacteria present in PM. Taken together, these results suggest that PM is more functionally similar to mammalian milk than was previously thought. PM and mammalian milk both have nutritional and immune modulatory components, and the ability to modulate the microbiota of the gut. This is fascinating from an evolutionary point of view when one considers that mammals and birds evolved these processes independently.

## Methods

### Ethics statement

All work using animals was conducted in accordance with the Australian Code of Practise for the Care and Use of Animals for Scientific Purposes (7^th^ edition), and in accordance with institutional animal ethics guidelines (Commonwealth Scientific and Industrial Research Organisation (CSIRO) Australian Animal Health Laboratory (AAHL) Animal Ethics Committee approval numbers 1289,1357 and 1446; and Deakin University Animal Ethics Committee approval numbers AEX56/2008 and AEX57/2008).

### Collection of pigeon ‘milk’

Breeding pairs of King pigeons were purchased from Kooyong Squab Producers (Moama, New South Wales, Australia) and housed in temperature controlled cabinets (between 21°C to 24°C) with a 12 hour light cycle (lights on 6 am). They were supplied with nest bowls and materials and had *ad libitum* access to pigeon mix (pro-vit-min, Ivorsons, Geelong, Australia) and water. Pigeons were allowed to breed, and were culled, along with their squabs, at either the time the squab hatched, or 2 days after the squab hatched. Pigeon ‘milk’ was collected from the crop of the parents and the squabs into sterile 2 mL tubes and frozen at −80°C until use. Samples were thawed at 4°C and pooled before use.

### Chicken husbandry

Sixteen newly hatched male Ross308 chickens were purchased from a commercial supplier (Bartter Enterprises, Bannockburn, Victoria, Australia). They were randomly assigned into 2 groups, wing-tagged for identification and weighed. The chicks were housed in separate cages within the same cabinet, to prevent access to the other group’s feed. Heat lamps were provided at one side of each cage to establish a temperature gradient. To keep the pigeon ‘milk’ fresh, the chicks were fed three times a day by mixing the pigeon ‘milk’ into a pre-weighed amount of antibiotic-free chicken feed (Country Heritage Feeds OPO05, Queensland, Australia), which was placed on a tray in the cage. Before each feed the amount of feed consumed by each group was calculated. Each chicken received on average 5 grams of pigeon ‘milk’ per day for 7 days.

A subsequent trial investigating the effect of the protein and fat components of pigeon ‘milk’ was set up as described above, where the replacement pigeon ‘milk’ consisted of peptone proteose (Becton Dickson, Australia) equivalent to 45% and pig lard (Fonterra, Australia) equivalent to 11%. These were chosen as they had the most similar amino acid and fatty acid compositions to pigeon ‘milk’.

### Chicken measurements and sample collection

Body mass of each chicken was determined on day 4. The chickens were culled after 7 days and their final weight was recorded. The following measurements were taken: from the top of the cranium to the cloaca (height), from the end of the furthermost wing digit on the left to the furthermost digit on the right (wing span), and from the patella to the posterior end of the tarsometatarsus (leg span). The breast muscle was removed from the breast bone with a scalpel and weighed. The caecal tonsils and ileum (adjacent to the caecal tonsils) were removed and collected in RNALater (Invitrogen) and frozen at −20°C until RNA extraction. The contents of the cecum was collected in sterile 5 mL containers and frozen at −20°C until DNA extraction.

### Statistical analysis of chicken body measurements

A statistical comparison of control and PM-fed chicken body measurements was performed with an unpaired t-test. Average percent body mass gain of PM-fed and PM replacement-fed chickens was calculated by normalising the weight gain of each experimental group chicken to the median weight gain of the corresponding control group chickens. A Kruskal-Wallis test with Dunns post-hoc test was used to identify any statistically significant difference in body mass gain between control, PM-fed and PM replacement-fed chickens.

### RNA isolation, labelling and microarray hybridisation

RNA was extracted from the caecal tonsil and ileum tissue of 6 control and 6 PM-fed chickens (mean weights) using a Cartagen RNA extraction kit (Inbio, Eltham, Australia) according to the manufacturer's instructions. cDNA was synthesised from 5 µg RNA using SuperScript III (Invitrogen) with oligo_dt_ primer. This was purified with a Qiagen PCR Purification Kit and labelled with Cy3 using a Roche One-Color DNA Labelling Kit according to the manufacturer's instructions. The labelled microarray probes were resuspended with a sample tracking control and hybridisation buffer and loaded on 12-plex 135 k custom chicken microarrays (NimbleGen design #10309). The array contains 65,850 probes printed in duplicate, of which there are 32,357 probes with unique UniGene IDs. Most unique genes have 2 or more probes. Information on the custom array is available from ArrayExpress using the accession number A-MEXP-2133. These were hybridised for 20 hours in a NimbleGen Hybridisation Station (Roche) at 42°C and then washed using the NimbleGen wash buffer kit (Roche) according to the manufacturer's instructions. Each subarray was scanned at 2 µm on autogain with a NimbleGen MS200 microarray scanner (Roche).

### Microarray quality control and statistical analysis

Sample tracking controls and control spots were used to autoalign a grid over each subarray using NimbleGen MS200 software (Roche), and Robust Multichip Average (RMA) analysis [51] was used to background correct and normalise the spot signal intensity. The datasets, along with probe annotation information, were exported into GeneSpring (Agilent) and differentially expressed genes were identified using Student's t-test, assuming unequal variances, with a false discovery rate of *p* = 0.05. Control ileum was compared to PM-fed ileum, and control caecal tonsil was compared to PM-fed caecal tonsil. All results have been deposited into the ArrayExpress database with accession number E-MTAB-1127.

### IgA expression analysis

The relative expression level of the IgA heavy chain (probe CLIGG_34917) was calculated from the RMA normalised spot signal intensity by dividing each probe by the total probe intensity and multiplying by 10 million. The relative signal intensity in the ileum and caecal tonsil for PM-fed chickens and control chickens was subjected to an unpaired t-test, and the mean and standard error of the mean was calculated and graphed using GraphPad5.

### Gene functional analysis

The DAVID functional annotation tool [52] was used to identify pathways and biological functions up-regulated in the caecal tonsil and ileum in association with pigeon ‘milk’. An ease score of 0.05 was used to determine enriched Kyoto Encyclopedia of Genes and Genomes (KEGG) pathways and Gene Ontology (GO) FAT biological functions.

Interferon-stimulated genes were functionally annotated using the Interferon Stimulated Gene Database [53] and/or a literature search.

### Caecal DNA extraction and 16S amplification

Total DNA was extracted from caecal contents as per the method of Yu and Morrison [54]. DNA quality and quantity was measured on a NanoDrop ND-1000 spectrophotometer. The V1-V3 region of bacterial 16S rRNA was amplified from caecal DNA following the method of Stanley *et al* using the primers and conditions previously detailed [55].

### High throughput 16S amplicon sequencing and data pre-processing

The amplified 16S rRNA gene samples from each bird were pooled using approximately equal amounts of each PCR product. The pooled sample was sequenced using the Roche/454 FLX Genome Sequencer and Titanium chemistry according to the manufacturer's instructions. Sff files were split into fasta and qual files using PyroBayes [56], and data was analysed with Qiime v1.3.0 software [57], except for OTU picking, denoising and chimera detection which was done using Otupipe [58]. Two samples (C1 and C3) were removed from analysis due to low sequence numbers per sample. Additional filtering of samples was performed to remove OTUs present in less than 3 samples or with less than 5 sequences. The default Qiime analysis parameters were used except as follows: sequence length 300–600 bases, no ambiguous sequences allowed, maximum of 6 homopolymers and classification by RDP. OTU sequences have been deposited in the European Molecular Biology Laboratory EMBL-Bank with accession numbers HE814242-HE814562.

### Network analysis of OTUs

Filtered, multiple rarefied OTU abundance data was used to generate a network of shared OTUs in Cytoscape v2.8.

### Analysis of bacteria that are differentially abundant in the cecum of PM-fed chickens and control chickens

Raw filtered OTU reads for each control chicken and PM-fed chicken sample were imported into Metastats [59] for statistical analysis, using 1000 permutations, to identify OTUs that were differentially abundant between control chickens and PM-fed chickens. OTUs were considered differentially abundant if the *p* value was less than 0.05.

### Identification of shared OTUs in PM, ctrl and PM-fed chickens

OTUs were called as present if the filtered, multiple rarefied count was greater than zero. For shared OTUs, the representative OTU sequence was uploaded to EZTaxon [60] and the closest cultured isolate was identified.

## Supporting Information

Figure S1 **Body mass gain of PM and PM replacement-fed chickens.** PM-fed chickens (n = 8) gained significantly more body mass than control chickens over 7 days. There was no difference between body mass gain of control chickens (n = 16) and PM-replacement-fed chickens (n = 8).
(JPG)

Figure S2 **UniFrac analysis of bacteria present in PM, PM-fed and control chickens.** Principal Coordinate Analysis plot based on unweighted UniFrac. Rarefied samples of PM are represented by yellow circles, PM-fed chickens by red triangles and control chickens by green squares.
(JPG)

Table S1 **Biological processes up-regulated in the gut of PM-fed chickens.** Gene ontology biological processes that were identified as enriched amongst genes up-regulated in ileum or caecal tonsil of PM-fed chickens (n = 6).
(DOCX)

Table S2 **Biological processes down-regulated in the gut of PM-fed chickens.** Gene ontology biological processes that were identified as enriched amongst genes down-regulated in ileum or caecal tonsil of PM-fed chickens (n = 6).
(DOCX)

Table S3 **Enriched KEGG pathways in the gut of PM-fed chickens.** KEGG pathways that were identified as enriched amongst differentially expressed genes in ileum or caecal tonsil of PM-fed chickens (n = 6).
(DOCX)
