# Supplementary figures and images for: Functional Similarities between Pigeon ‘Milk’ and Mammalian Milk: Induction of Immune Gene Expression and Modification of the Microbiota
Source: PLoS One. 2012 Oct 26;7(10):e48363. doi: 10.1371/journal.pone.0048363 (PMC3482181; doi:10.1371/journal.pone.0048363)

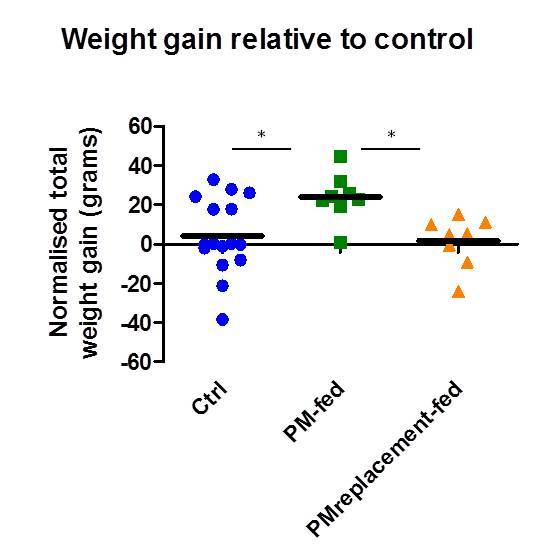

Supplement: Figure S1 — Body mass gain of PM and PM replacement-fed chickens. PM-fed chickens (n = 8) gained significantly more body mass than control chickens over 7 days. There was no difference between body mass gain of control chickens (n = 16) and PM-replacement-fed chickens (n = 8). (JPG) [file pone.0048363.s001.jpg]

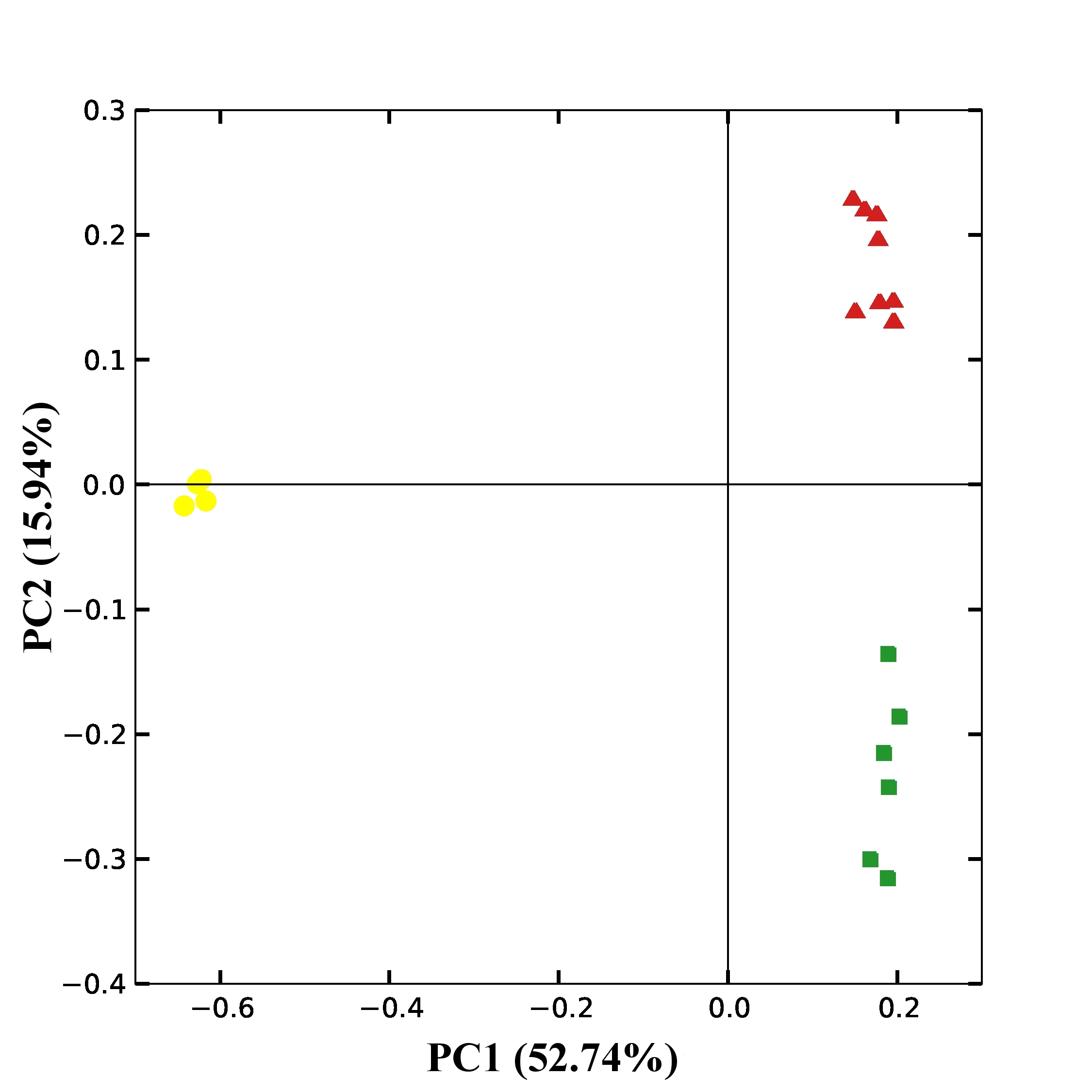

Supplement: Figure S2 — UniFrac analysis of bacteria present in PM, PM-fed and control chickens. Principal Coordinate Analysis plot based on unweighted UniFrac. Rarefied samples of PM are represented by yellow circles, PM-fed chickens by red triangles and control chickens by green squares. (JPG) [file pone.0048363.s002.jpg]
