# Supplementary material for: Functional Similarities between Pigeon ‘Milk’ and Mammalian Milk: Induction of Immune Gene Expression and Modification of the Microbiota
Source: PLoS One. 2012 Oct 26;7(10):e48363. doi: 10.1371/journal.pone.0048363 (PMC3482181; doi:10.1371/journal.pone.0048363)
Supplement: Table S1 — Biological processes up-regulated in the gut of PM-fed chickens. Gene ontology biological processes that were identified as enriched amongst genes up-regulated in ileum or caecal tonsil of PM-fed chickens (n = 6). (DOCX) [file pone.0048363.s003.docx]

## Table S1 - Biological processes up-regulated in the gut of PM-fed chickens

| GO FAT Biological process | %Total genes^ | %Assigned GO ID* | *p* value |
| --- | --- | --- | --- |
| **Ileum** |  |  |  |
| GO:0045087 innate immune response | 1.15 | 16.66 | 2.0e-2 |
| GO:0035556 intracellular signalling cascade | 4.61 | 66.66 | 2.4e-2 |
| GO:0001817 regulation of cytokine production | 1.44 | 20.83 | 3.0e-2 |
| GO:0050871 positive regulation of B cell activation | 0.86 | 12.5 | 3.1e-2 |
| **Cecal tonsil** |  |  |  |
| GO:0050864 regulation of B cell activation | 2.20 | 16 | 3.94e-4 |
| GO:0002683 negative regulation of immune system process | 2.20 | 16 | 4.89e-4 |
| GO:0050869 negative regulation of B cell activation | 1.65 | 12 | 6.73e-4 |
| GO:0001775 cell activation | 3.30 | 24 | 2.03e-3 |
| GO:0002695 negative regulation of leukocyte activation | 1.65 | 12 | 3.05e-3 |
| GO:0051250 negative regulation of lymphocyte activation | 1.65 | 12 | 3.05e-3 |
| GO:0030888 regulation of B cell proliferation | 1.65 | 12 | 3.90e-3 |
| GO:0050866 negative regulation of cell activation | 1.65 | 12 | 4.84e-3 |
| GO:0006955 immune response | 3.30 | 24 | 6.68e-3 |
| GO:0051249 regulation of lymphocyte activation | 2.20 | 16 | 1.01e-2 |
| GO:0030098 lymphocyte differentiation | 2.20 | 16 | 1.07e-2 |
| GO:0002694 regulation of leukocyte activation | 2.20 | 16 | 1.21e-2 |
| GO:0050865 regulation of cell activation | 2.20 | 16 | 1.53e-2 |
| GO:0030097 hemopoiesis | 2.75 | 20 | 1.54e-2 |
| GO:0042981 regulation of apoptosis | 3.85 | 28 | 1.74e-2 |
| GO:0043067 regulation of programmed cell death | 3.85 | 28 | 1.90e-2 |
| GO:0010941 regulation of cell death | 3.85 | 28 | 1.94e-2 |
| GO:0002521 leukocyte differentiation | 2.20 | 16 | 2.08e-2 |
| GO:0033673 negative regulation of kinase activity | 1.65 | 12 | 2.10e-2 |
| GO:0006469 negative regulation of protein kinase activity | 1.65 | 12 | 2.10e-2 |
| GO:0030889 negative regulation of B cell proliferation | 1.10 | 8 | 2.14e-2 |
| GO:0002902 regulation of B cell apoptosis | 1.10 | 8 | 2.14e-2 |
| GO:0045736 negative regulation of cyclin-dependent protein kinase activity | 1.10 | 8 | 2.14e-2 |
| GO:0048534 hemopoietic or lymphoid organ development | 2.75 | 20 | 2.26e-2 |
| GO:0051348 negative regulation of transferase activity | 1.65 | 12 | 2.49e-2 |
| GO:0002520 immune system development | 2.75 | 20 | 2.65e-2 |
| GO:0070663 regulation of leukocyte proliferation | 1.65 | 12 | 2.91e-2 |
| GO:0050670 regulation of lymphocyte proliferation | 1.65 | 12 | 2.91e-2 |
| GO:0032944 regulation of mononuclear cell proliferation | 1.65 | 12 | 2.91e-2 |
| GO:0050672 negative regulation of lymphocyte proliferation | 1.10 | 8 | 3.20e-2 |
| GO:0070228 regulation of lymphocyte apoptosis | 1.10 | 8 | 3.20e-2 |
| GO:0070664 negative regulation of leukocyte proliferation | 1.10 | 8 | 3.20e-2 |
| GO:0032945 negative regulation of mononuclear cell proliferation | 1.10 | 8 | 3.20e-2 |
| GO:0046649 lymphocyte activation | 2.20 | 16 | 3.22e-2 |
| GO:0042325 regulation of phosphorylation | 2.75 | 20 | 3.53e-2 |
| GO:0019220 regulation of phosphate metabolic process | 2.75 | 20 | 4.03e-2 |
| GO:0051174 regulation of phosphorus metabolic process | 2.75 | 20 | 4.03e-2 |
| GO:0030217 T cell differentiation | 1.65 | 12 | 4.33e-2 |
| GO:0009057 macromolecule catabolic process | 3.30 | 24 | 4.38e-2 |
| GO:0045321 leukocyte activation | 2.20 | 16 | 4.47e-2 |

Gene ontology biological processes that were identified as enriched amongst genes up-regulated in ileum or cecal tonsil of PM-fed chickens using an ease score cut-off of 0.05.

^Total genes is the number of up-regulated genes (ileum=347, cecal tonsil = 182) that could be identified by the DAVID program.

*Assigned GO ID is the number of genes (ileum = 24, cecal tonsil = 25) that could be assigned a GO ID.
