# Supplementary material for: Functional Similarities between Pigeon ‘Milk’ and Mammalian Milk: Induction of Immune Gene Expression and Modification of the Microbiota
Source: PLoS One. 2012 Oct 26;7(10):e48363. doi: 10.1371/journal.pone.0048363 (PMC3482181; doi:10.1371/journal.pone.0048363)
Supplement: Table S2 — Biological processes down-regulated in the gut of PM-fed chickens. Gene ontology biological processes that were identified as enriched amongst genes down-regulated in ileum or caecal tonsil of PM-fed chickens (n = 6). (DOCX) [file pone.0048363.s004.docx]

## Table S2 - Biological processes down-regulated in the gut of PM-fed chickens

| Pathway | %Total genes^ | %Mapped genes* | *p* value |
| --- | --- | --- | --- |
| **Ileum** |  |  |  |
| GO:0051301 cell division | 3.24 | 25 | 3.53e-03 |
| GO:0022402 cell cycle process | 3.78 | 29.16 | 9.89e-03 |
| GO:0007049 cell cycle | 4.32 | 33.33 | 1.50e-02 |
| GO:0000279 M phase | 2.70 | 20.83 | 1.94e-02 |
| GO:0007067 mitosis | 2.16 | 16.66 | 2.88e-02 |
| GO:0000280 nuclear division | 2.16 | 16.66 | 2.88e-02 |
| GO:0048285 organelle fission | 2.16 | 16.66 | 3.33e-02 |
| GO:0000087 M phase of mitotic cell cycle | 2.16 | 16.66 | 3.33e-02 |
| GO:0016569 covalent chromatin modification | 2.16 | 16.66 | 3.57e-02 |
| GO:0006916 anti-apoptosis | 2.16 | 16.66 | 3.81e-02 |
| GO:0043066 negative regulation of apoptosis | 3.24 | 25 | 4.24e-02 |
| GO:0006457 protein folding | 2.70 | 20.83 | 4.28e-02 |
| GO:0043069 negative regulation of programmed cell death | 3.24 | 25 | 4.55e-02 |
| GO:0060548 negative regulation of cell death | 3.24 | 25 | 4.55e-02 |
| GO:0022403 cell cycle phase | 2.70 | 20.83 | 4.67e-02 |
| GO:0051276 chromosome organization | 3.78 | 29.16 | 5.00e-02 |
| **Cecal tonsil** |  |  |  |
| GO:0008654 phospholipid biosynthetic process | 1.55 | 36.36 | 9.24e-03 |
| GO:0006497 protein amino acid lipidation | 1.16 | 27.27 | 1.39e-02 |
| GO:0042158 lipoprotein biosynthetic process | 1.16 | 27.27 | 1.76e-02 |
| GO:0008610 lipid biosynthetic process | 2.33 | 54.54 | 2.61e-02 |
| GO:0006576 biogenic amine metabolic process | 1.55 | 36.36 | 3.21e-02 |
| GO:0042157 lipoprotein metabolic process | 1.16 | 27.27 | 4.12e-02 |

Gene ontology biological processes that were identified as enriched amongst genes down-regulated in ileum or cecal tonsil of PM-fed chickens using an ease score cut-off of 0.05.

^Total genes is the number of down-regulated genes (ileum=185, cecal tonsil = 257) that could be identified by the DAVID program.

*Assigned GO ID is the number of genes (ileum = 24, cecal tonsil = 11) that could be assigned a GO ID.
