# Supplementary material for: Functional Similarities between Pigeon ‘Milk’ and Mammalian Milk: Induction of Immune Gene Expression and Modification of the Microbiota
Source: PLoS One. 2012 Oct 26;7(10):e48363. doi: 10.1371/journal.pone.0048363 (PMC3482181; doi:10.1371/journal.pone.0048363)
Supplement: Table S3 — Enriched KEGG pathways in the gut of PM-fed chickens. KEGG pathways that were identified as enriched amongst differentially expressed genes in ileum or caecal tonsil of PM-fed chickens (n = 6). (DOCX) [file pone.0048363.s005.docx]

## Table S3 - Enriched KEGG pathways in the gut of PM-fed chickens

| Pathway | %Total genes^ | %Mapped genes* | *p* value |
| --- | --- | --- | --- |
| **Ileum – up-regulated** |  |  |  |
| Jak-STAT signalling pathway | 2.59 | 31.03 | 5.6e-3 |
| Natural killer cell mediated cytotoxicity | 2.01 | 24.13 | 6.0e-3 |
| Toll-like receptor signalling pathway | 2.01 | 24.13 | 1.4e-2 |
| Cytokine-cytokine receptor interaction | 2.59 | 31.03 | 1.8e-2 |
| Lysosome | 2.01 | 24.13 | 3.8e-2 |
| Progesterone-mediated oocyte maturation | 1.72 | 20.68 | 3.9e-2 |
| **Cecal tonsil – up-regulated** |  |  |  |
| Cytokine-cytokine receptor interaction | 3.30 | 100 | 2.6e-2 |
| **Cecal tonsil – down-regulated** |  |  |  |
| Regulation of actin cytoskeleton |  |  |  |
| Spliceosome |  |  |  |

KEGG pathways that were identified as enriched amongst differentially expressed genes in ileum or cecal tonsil of PM-fed chickens using an ease score of 0.05. There were no enriched KEGG pathways amongst genes down-regulated in ileum of PM-fed chickens.

^Total genes is the number of up or down-regulated genes (ileum = 347, cecal tonsil up-regulated= 182, cecal tonsil down-regulated = 257) that could be identified by the DAVID program.

*Mapped genes is the number of genes (ileum = 29, cecal tonsil up-regulated = 6, cecal tonsil down-regulated = 14) that could be mapped to a KEGG pathway. Genes can be mapped to multiple pathways.
